# Supplementary material for: What delirium detection tools are used in routine clinical practice in the United Kingdom? Survey results from 91% of acute healthcare organisations
Source: Eur Geriatr Med. 2021 May 18;12(6):1293–8. doi: 10.1007/s41999-021-00507-2 (PMC8626368; doi:10.1007/s41999-021-00507-2)
Supplement: Supplementary file 1 — Supplementary file1 (DOCX 17 kb) [file 41999_2021_507_MOESM1_ESM.docx]

Article title: What delirium detection tools are used in routine clinical practice in the UK?

A Freedom of Information investigation of UK hospitals.

Journal: European Geriatric Medicine

Authors: Zoë Tieges^1*^, Jacqueline Lowrey^2^, Alasdair M. J. MacLullich^1^

Affiliations: ^1^Edinburgh Delirium Research Group, Geriatric Medicine, Usher Institute, University of Edinburgh, Edinburgh, Scotland, UK; ^2^College of Medicine and Veterinary Medicine, Queen's Medical Research Institute, University of Edinburgh, Edinburgh, Scotland, UK.

*Corresponding author: zoe.tieges@ed.ac.uk.

Supplementary File 1: Freedom of Information questions.

1.   Do you use a delirium assessment tool as part of clinical practice for your non-ICU patients in your trust/hospital?  YES / NO

2.   If yes, in which clinical settings are they in place (please use X to indicate all that apply)?

_____ Acute general medicine/Medicine of the Elderly

_____ Emergency Department

_____ Surgical wards

_____ Other (please specify):

3.   Which, if any, validated tools are included in your written (paper or electronic) policies? Please use X to indicate all that apply.

_____ 4 ‘A’s Test (4AT)

_____ Confusion Assessment Method

_____ Single Question in Delirium

_____ Other (please specify):

4.   Do you have a pathway or guidelines relating to delirium?  YES / NO

If yes, in which year were they written? Please attach an electronic copy.
